# Associations of serum multivitamin levels with the risk of non-alcoholic fatty liver disease: A population-based cross-sectional study in U.S. adults

**DOI:** 10.3389/fnut.2022.962705

**Published:** 2022-09-12

**Authors:** Hongye Peng, Miyuan Wang, Liang Pan, Zhengmin Cao, Ziang Yao, Qiuye Chen, Yanbo Li, Yuhua Wang, Wenliang Lv

**Affiliations:** ^1^Department of Infection, Guang’anmen Hospital, China Academy of Chinese Medical Sciences, Beijing, China; ^2^School of Public Health, Tongji Medical College, Huazhong University of Science and Technology, Wuhan, China; ^3^Phase 1 Clinical Trial Center, Deyang People’s Hospital, Deyang, China

**Keywords:** multi-vitamins, NAFLD, weighted quantile sum, dose-response relationship, serum level

## Abstract

Vitamins were closely associated with non-alcoholic fatty liver disease (NAFLD) development, but no study had explored the association of serum multivitamin levels with NAFLD risk. We assessed the association between serum levels of both single-vitamin and multivitamins (VA, VB6, VB9, VB12, VC, VD, and VE) and the risk of NAFLD, using the database of National Health and Nutrition Examination Survey (NHANES) (cycles 2003–2004 and 2005–2006). We employed multivariable logistic regression and weighted quantile sum (WQS) regression models to explore the association of serum multivitamin levels with NAFLD. Among all 2,294 participants, 969 participants with NAFLD were more likely to be male, older, less educated, or have hypertension/high cholesterol/diabetes. After adjustment of covariates, serum VC/VD/VB6/VB9 levels were negatively correlated with NAFLD risk, while serum VA/VE levels were positively correlated with NAFLD risk. In the WQS model, elevated serum VA/VE levels and lowered serum VC/VD/VB6 levels were linearly associated with increased NAFLD risk. There was a non-linear relationship between serum VB9/VB12 levels and NAFLD risk. There were evident associations between serum multivitamin levels and reduced NAFLD risk, which was mainly driven by VD/VB9/VC. In conclusion, our findings suggested that serum multivitamin levels were significantly associated with the risk of NAFLD.

## Introduction

Non-alcoholic fatty liver disease (NAFLD) is a syndrome characterized by excessive deposition of fat in hepatocytes other than factors from alcohol or other definite liver damage, encompassing a spectrum of non-alcoholic fatty liver, non-alcoholic steatohepatitis, related cirrhosis, liver cancer, and others. NAFLD has become the most common chronic liver disease worldwide and exerts influence on the health of 25.24% adults (1). With increasing living standards and obese population, the prevalence of NAFLD will rise continuously. In 2030, the prevalence of NAFLD is expected to reach 33.5% in population aged over 15 years and 28.4% in all ages, with 100.9 million newly increased cases (2). The increasing population with NAFLD imposes a growing societal and economic burden to the world. Presently, there is a lack of safe and effective drug for NAFLD, and the most effective way is lifestyle improvement (3). It is noteworthy that further research studies should focus on the prevention and relief of NAFLD development.

As a class of organic compounds necessary for maintaining the normal physiological function, vitamins play an important role in the growth, metabolism, and development of human body. More and more evidences suggest that vitamins are closely associated with the occurrence and development of NAFLD (4–7). However, some results are controversial. For example, individuals with elevated serum VB9 and VB12 levels have lower risk of NAFLD (8, 9), while those with elevated serum VA level possibly have higher risk of NAFLD (10). Most studies currently focus on the relationship between serum level of single-vitamin, instead of multivitamins, and the development of NAFLD. Previous studies demonstrate that some vitamins may affect the biological functions by interacting with other vitamins (11). So it is significant to explore the potential association between serum multivitamin levels and NAFLD risk.

The absorption of vitamins is affected by various factors, including gender, age, and BMI (12). And also there is vitamin loss during food storage, processing, and cooking. Therefore, the serum vitamin level is a more representative and persuasive indicator to evaluate vitamin circulation, comparing with vitamin intake from food.

Based on the data from 2003 to 2006 in the National Health and Nutrition Examination Survey (NHANES), this study aims to elaborate the relationship between the serum levels of 7 vitamins (VA, VB6, VB9, VB12, VC, VD, and VE) and the risk of NAFLD in American adults, as well as to explore the association between serum levels of both single-vitamin and multivitamins and the prevalence of NAFLD.

## Materials and methods

### Study design and participants

**FIGURE 1:**
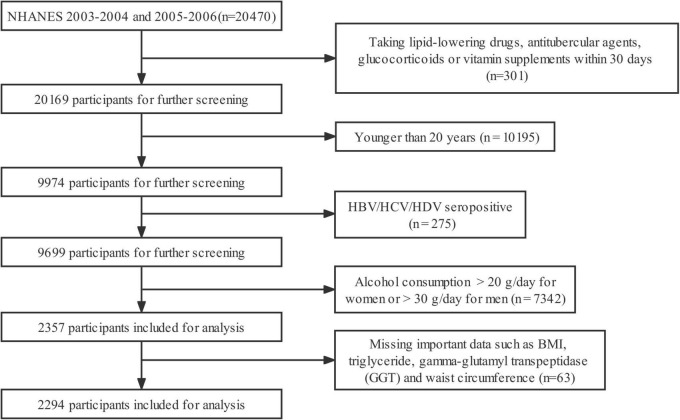
Flow chart for the selection of participants in the cohort study. HBV, hepatitis B virus; HCV, hepatitis C virus; HDV, hepatitis D virus.

This study included all participants ≥ 20 years old from the 2003–2004 and 2005–2006 cycles of the NHANES in the United States. Profiles of the NHANES were introduced by other researchers (13). Employing a complex, multi-stage, and probability sampling design, the NHANES collected a representative sample from non-institutionalized American population. In those two cycles of NHANES, all baseline data were collected by household interviews, mobile physical examinations, and laboratory tests. Figure 1 showed the flow chart of participant screening. Of all the 20,470 participants, we excluded those taking lipid-lowering drugs, antitubercular agents, glucocorticoids, or vitamin supplements within 30 days (*n* = 301), those < 20 years old (*n* = 10,195), those with positive serology for hepatitis B, C, and D (*n* = 275), those consuming alcohol > 20 g/day for female or > 30 g/day for male (*n* = 7,342), and those missing important data such as BMI, triglyceride, gamma-glutamyl transpeptidase (GGT), and waist circumference (*n* = 63) (Figure 1). At last, a total of 2,294 participants were included for the statistical analysis. The protocol of NHANES was reviewed and approved by the Research Ethics Review Board of the National Center for Health Statistics. All participants signed written informed consent before their participation.

### Definition of non-alcoholic fatty liver disease

NAFLD was a condition of excessive fat accumulation in the liver, excluding fatty liver due to other causes by liver disease and/or excessive alcohol consumption. Fatty liver index (FLI) was considered a simple and accurate predicting index for hepatic steatosis in the general population and was widely used in NAFLD research, with an accuracy of 0.84 (95% CI: 0.81–0.87) (14). So we used FLI to predict the risk of NAFLD. FLI was calculated with the following Equation (15):


$$\text{FLI}=({\text{e}}^{0.\text{953}\times \text{LN }\left(\text{triglycerides}\right)\;+\;0.\text{139}\times \text{BMI}+0.\text{718}\times \text{LN }\left(\text{GGT}\right)}$$

$${}^{+0.0\text{53}\times \text{waist circumference}-\text{15}.\text{745}})÷(\text{1}+{\text{e}}^{0.\text{953}\times \text{LN }\left(\text{triglycerides}\right)}$$

)+0.139×BMI+0.718×LN⁢(GGT)+0.053×waistcircumference-15.745×100,

where triglycerides were in mg/dl, BMI in kg/m^2^, GGT in mmol/L, and waist circumference in cm. This equation of FLI generated scores ranging from 0 to 100. An FLI < 30 suggested no presence of NAFLD, while an FLI ≥ 60 suggested the probable presence of fatty liver (15).

### Measurement of serum vitamin levels

All serum samples were processed and delivered to the Division of Environmental Health Laboratory Sciences, National Center for Environmental Health, and Centers for Disease Control and Prevention (CDC) for analysis. Serum levels of VA (retinol), VC (ascorbic acid), and VE (α-tocopherol) were measured with high-performance liquid chromatography (HPLC) and quantitatively determined with spectrophotometric methods. Serum 25-hydroxyvitamin D [25(OH)D] levels were first determined with the DiaSorin RIA kit. NHANES employed regression to convert equivalently all 25(OH)D measurements obtained from standardized liquid chromatography–tandem mass spectrometry (LC-MS/MS) methods, to integrate the laboratory test data from different cycles. Serum VB9 and VB12 levels were measured with the radio-assay kit from Bio-Rad Laboratories. Serum VB6 level was measured with reversed-phase HPLC methods. All the laboratory assay data were accessible to the public on the NHANES website.

### Covariates

Demographic variables were extracted from the baseline household questionnaires as covariates, including age, gender, ethnicity (Mexican American, non-Hispanic white, non-Hispanic black, and others), family income—poverty ratio (FIPR), and education level (below high school, high school or equivalent, and college or above). Histories of hypertension, high cholesterol, and diabetes were also collected. More details of the above-mentioned covariates were accessible to the public on the NHANES website.

### Statistical analysis

According to the analytic guidelines for the NHANES survey, we employed sampling weights in our analyses and estimated variances by considering clustering and stratification. Binary or categorical variables were presented with number (%), while continuous variables were presented with median (interquartile range, IQR). The differences of population characteristics within the survey cycle were analyzed with Rao–Scott Chi-square test and Wilcoxon rank-sum test for categorical and continuous variables, respectively.

Multivariate-adjusted logistic regression models were employed to explore the relationship between the serum level of single vitamin and NAFLD. Serum levels of vitamins were analyzed as both continuous and categorical variables (classified into four groups according to quartiles, with the first quartile as reference). We computed the odds ratio (OR) and corresponding 95% confidence intervals (95% CIs) in three models. Model 1 included only independent variables. Model 2 was additionally adjusted for gender, age, ethnicity, FIPR, and education level. Model 3 was further adjusted for the disease history (hypertension, high cholesterol, and diabetes).

Weighted Quantile Sum (WQS) regression, a weighted quartile sum approach combined with logistic regression, was employed to examine the associations between both multivitamins/single-vitamin and the risk of NAFLD. The WQS regression integrated the serum levels of multivitamins into one index. The contribution of a single vitamin level was weighted according to its relevance to the overall association with the outcome. The weights are constrained to sum to 1, with higher numbers indicating a larger contribution (16, 17). The serum levels of vitamins were highly correlated, so there would be misleading results when employing linear regression models to examine the association of a single vitamin level with NAFLD while adjusting for other levels, due to the problem of collinearity. Therefore, we proposed WQS regression to examine the association of single serum vitamin level with NAFLD, while addressing the highly positive correlations among multivitamin levels.

All statistical analyses were completed with R 3.6.2, employing packages “lme4”. *p* < 0.05 for a two-tailed test denoted statistical significance.

## Results

### Characteristics of the study participants

There were 969 individuals with NAFLD (42.24%) among the 2,294 participants (Table 1). The mean ± SD age was 51 (36, 67) years [54 (40, 67) in participants with NAFLD and 49 (34, 67) in those without]. The proportions of men and women were 56.8 and 43.2%, respectively. Except for ethnicity and FIPR, there were statistical differences between participants with and without NAFLD in the basic characteristics. Participants with NAFLD were more likely to be male, older, less educated, or have hypertension/high cholesterol/diabetes. Most serum vitamin levels were lower in participants with NAFLD than in those without, except for VA and VE (Table 1).

**TABLE 1:** Basic characteristics of participants by non-alcoholic fatty liver disease (NAFLD) in NHANES 2003–2006.

Variables	Non-NAFLD (*n* = 1325)	With NAFLD (*n* = 969)	Total (*n* = 2294)	*P-*value
**Sex**, ***n* (%)**				<0.001
Female	650 (49.1)	341 (35.2)	991 (43.2)	
Male	675 (50.9)	628 (64.8)	1303 (56.8)	
Age (years)	49 (34, 67)	54 (40, 67)	51 (36, 67)	<0.001
**Race**, ***n* (%)**				0.431
Mexican American	178 (13.4)	154 (15.9)	332 (14.5)	
Non-Hispanic black	225 (17)	158 (16.3)	383 (16.7)	
Non-Hispanic white	847 (63.9)	603 (62.2)	1450 (63.2)	
Other	75 (5.7)	54 (5.6)	129 (5.6)	
FIPR	3.3 (1.7, 5)	3 (1.6, 5)	3.2 (1.7, 5)	0.082
Missing	49 (3.7%)	37 (3.8%)	86 (3.7%)	
**Education**, ***n* (%)**				0.006
College or above	828 (62.5)	542 (55.9)	1370 (59.7)	
High school or equivalent	277 (20.9)	236 (24.4)	513 (22.4)	
Less than high school	219 (16.5)	191 (19.7)	410 (17.9)	
Missing	1 (0.1%)	0 (0%)	1 (0.0%)	
**Hypertension**, ***n* (%)**				<0.001
No	980 (74.2)	518 (53.8)	1498 (65.6)	
Yes	341 (25.8)	444 (46.2)	785 (34.4)	
Missing	4 (0.3%)	7 (0.7%)	11 (0.5%)	
**High cholesterol**, ***n* (%)**				<0.001
No	620 (62.2)	389 (48.9)	1009 (56.3)	
Yes	376 (37.8)	407 (51.1)	783 (43.7)	
Missing	329 (24.8%)	173 (17.9%)	502 (21.9%)	
**Diabetes**, ***n* (%)**				<0.001
No	1259 (95.8)	806 (85.3)	2065 (91.4)	
Yes	55 (4.2)	139 (14.7)	194 (8.6)	
Missing	11 (0.8%)	24 (2.5%)	35 (1.5%)	
VA (μmol/L)	2.0 (1.7, 2.4)	2.2 (1.8, 2.6)	2.1 (1.7, 2.5)	<0.001
Missing	11 (0.8%)	8 (0.8%)	19 (0.8%)	
VE (μmol/L)	27.9 (22.3, 35.8)	30.1 (24.1, 40.6)	28.7 (22.9, 37.8)	<0.001
Missing	11 (0.8%)	16 (1.7%)	27 (1.2%)	
VD (μmol/L)	62.4 (48.3, 75.3)	56.8 (42.2, 68.9)	59.2 (45.9, 72.9)	<0.001
Missing	3 (0.2%)	1 (0.1%)	4 (0.2%)	
VC (μmol/L)	63.0 (48.3, 77.2)	51.7 (34, 67.7)	59.1 (41.4, 73.8)	<0.001
Missing	7 (0.5%)	5 (0.5%)	12 (0.5%)	
VB6 (μmol/L)	53.3 (29.2, 93.1)	43.6 (26.9, 77)	49.5 (27.9, 85.7)	<0.001
Missing	41 (3.1%)	26 (2.7%)	67 (2.9%)	
VB12 (μmol/L)	366.0 (279.3, 493.0)	331.0 (250.2, 439.3)	352.0 (265.7, 467.3)	<0.001
Missing	17 (1.3%)	13 (1.3%)	30 (1.3%)	
VB9 (μmol/L)	30.8 (22, 41.7)	26.7 (19, 39.2)	29 (20.6, 40.6)	<0.001
Missing	8 (0.6%)	11 (1.1%)	19 (0.8%)	

FIPR, family income-poverty ratio; VA, vitamin A; VE, vitamin E; VC, vitamin C; VD, vitamin D; VB6, vitamin B6, VB12, vitamin B12; VB9, vitamin B9. P-values were calculated by Rao–Scott chi-square test and Wilcoxon rank-sum test for categorical and continuous variables, respectively.

**FIGURE 2:**
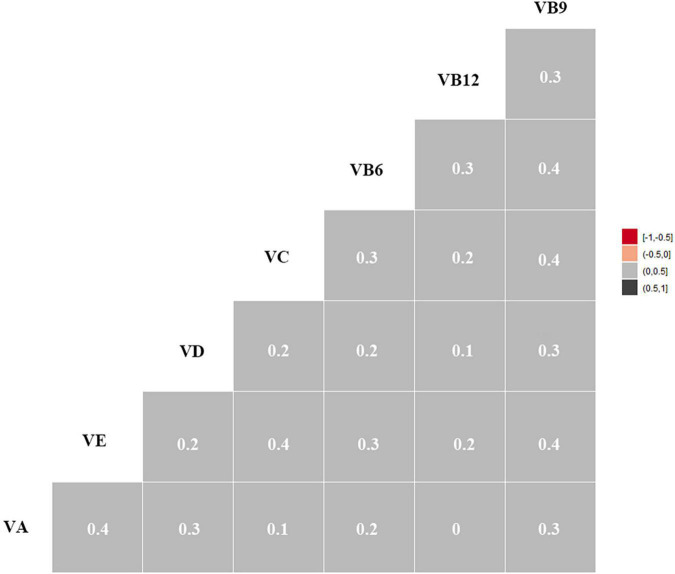
Heat Map for Spearman correlation between serum vitamins. Correlation intensity between two vitamins was indicated by square color. In the heat map, the black color shows a strong positive expression correlation, whereas the gray color depicts a weak expression correlation. VA, vitamin A; VE, vitamin E; VC, vitamin C; VD, vitamin D; VB6, vitamin B6, VB12, vitamin B12; VB9, vitamin B9.

Pearson correlations (rs) of all studied vitamins are shown in Figure 2. Most of the vitamins were positively correlated with each other (rs = 0.1–0.4). The correlation coefficients between VB6 and VB9, VC and VB9, VE and VC, VE and VB9, and VA and VE were 0.4.

### Serum level of single vitamin and the risk of non-alcoholic fatty liver disease

Table 2 showed the multi-variate adjusted OR and 95% CIs for the relationship between the risk of NAFLD and increment of serum level of seven studied vitamins. After adjusting for all covariates, negative associations with the risk of NAFLD were found for VC (OR = 0.985, 95% CI = 0.981–0.989), VD (OR = 0.981, 95% CI = 0.975–0.987), VB6 (OR = 0.995, 95% CI = 0.994–0.997), and VB9 (OR = 0.988, 95% CI = 0.979–0.997), while positive associations were found for VA (OR = 1.361, 95% CI = 1.045–1.772) and VE (OR = 1.020, 95% CI = 1.012–1.028). Moreover, we found that the risk of NAFLD increased by 36.1% (95% CI: 4.5%, 77.2%) with each unit increment of serum VA level in model 3.

**TABLE 2:** Multi-variate adjusted odds ratio (95% CIs) for the relationship between the risk of NAFLD and increment of serum level of vitamins among participants in NHANES 2003–2006.

Variables	Model l	Model 2	Model 3
VA	**1.768 (1.427, 2.189)**	**1.551 (1.226, 1.964)**	**1.361 (1.045, 1.772)**
VE	**1.017 (1.011, 1.024)**	**1.019 (1.011, 1.027)**	**1.020 (1.012, 1.028)**
VC	**0.981 (0.977, 0.985)**	**0.982 (0.978, 0.985)**	**0.985 (0.981, 0.989)**
VD	**0.985 (0.980, 0.990)**	**0.981 (0.976, 0.985)**	**0.981 (0.975, 0.987)**
VB6	**0.996 (0.994, 0.998)**	**0.995 (0.994, 0.997)**	**0.995 (0.994, 0.997)**
VB12	**0.999 (0.998, 1.000)**	**0.999 (0.998, 1.000)**	0.999 (0.998, 1.000)
VB9	**0.987 (0.979, 0.996)**	**0.985 (0.977, 0.994)**	**0.988 (0.979, 0.997)**

All estimates accounted for complex survey designs. Model 1 included only independent variables; model 2 was additionally adjusted for gender, age, ethnicity, FIPR and education level; and model 3 was further adjusted for the disease history (hypertension, high cholesterol and diabetes). Those results in bold had statistical significance.

We repeated the above-mentioned statistical analyses by using vitamins as categorical variables (quartiles) and acquired similar results (Table 3). In addition, the risk of NAFLD showed a stepwise decrease with the quartiles of VB9 (*P* for trend < 0.05) and was at the lowest point in the highest VB9 quartile group (Q4) (OR, 0.537; 95% CI, 0.364-0.793) after adjusting for all covariates. Similar trends were found for VC and VB6. After adjusting for all covariates in model 3, the risk of NAFLD was higher in Q4 group than that in the Q1 group for both serum VA (OR, 1.661 (1.093, 2.526)) and serum VE (OR, 1.976 (1.377, 2.836)).

**TABLE 3:** Multi-variate adjusted odds ratios (95% CIs) of risks of NAFLD in relation to serum vitamins levels among participants in NHANES 2003–2006.

Variables	The range of serum vitamin levels (μ mol/L)	Model 1	*P-*value	Model 2	*P-*value	Model 3	*P-*value
VA	0.34–4.78						
Q1	0.34–1.72	1		1		1	
Q2	1.72–2.07	**1.614 (1.239, 2.103)**	0.001	**1.441 (1.097, 1.892)**	0.017	1.35 (0.932, 1.955)	0.132
Q3	2.07–2.45	**1.619 (1.217, 2.155)**	0.003	1.248 (0.882, 1.766)	0.226	1.083 (0.715, 1.64)	0.712
Q4	2.45–4.78	**2.409 (1.761, 3.296)**	0.000	**1.934 (1.359, 2.752)**	0.002	**1.661 (1.093, 2.526)**	0.030
*p* for trend		<0.001		0.010			0.102
VE	2.04–89.12						
Q1	2.04–22.87	1		1		1	
Q2	22.89–28.68	1.185 (0.868, 1.619)	0.295	1.248 (0.893, 1.745)	0.210	1.393 (0.929, 2.09)	0.128
Q3	28.72–37.76	**1.397 (1.049, 1.861)**	0.030	**1.447 (1.043, 2.009)**	0.039	1.5 (1.014, 2.219)	0.059
Q4	37.83–89.12	**1.736 (1.303, 2.314)**	0.001	**1.905 (1.361, 2.666)**	0.001	**1.976 (1.377, 2.836)**	0.002
*p* for trend		<0.001		0.002		0.003	
VC	0.60–195.90						
Q1	0.60–41.40	1		1		1	
Q2	42.00–59.10	**0.585 (0.431, 0.795)**	0.002	**0.602 (0.437, 0.829)**	0.006	0.713 (0.498, 1.021)	0.083
Q3	59.60–73.80	**0.35 (0.262, 0.469)**	0.000	**0.374 (0.277, 0.505)**	0.000	**0.463 (0.338, 0.632)**	0.000
Q4	74.40–195.90	**0.301 (0.229, 0.396)**	0.000	**0.314 (0.246, 0.4)**	0.000	**0.377 (0.282, 0.503)**	0.000
*p* for trend		<0.001		<0.001		<0.001	
VD	9.10–166.00						
Q1	9.10–45.90	1		1		1	
Q2	47.10–59.20	0.951 (0.688, 1.314)	0.763	0.873 (0.603, 1.263)	0.479	0.987 (0.65, 1.499)	0.953
Q3	60.60–72.90	0.747 (0.562, 0.993)	0.055	**0.641 (0.475, 0.867)**	0.009	0.823 (0.552, 1.226)	0.352
Q4	73.80–166.00	**0.496 (0.372, 0.661)**	0.000	**0.411 (0.31, 0.546)**	0.000	**0.426 (0.286, 0.635)**	0.001
*p* for trend		<0.001		<0.001		<0.001	
VB6	3.80–400.00						
Q1	3.80–27.90	1		1		1	
Q2	28.00–49.50	1.136 (0.904, 1.427)	0.285	0.993 (0.786, 1.256)	0.955	0.871 (0.627, 1.211)	0.424
Q3	49.70–85.60	0.868 (0.672, 1.121)	0.288	0.741 (0.558, 0.985)	0.053	0.68 (0.472, 0.979)	0.055
Q4	85.80–400.00	**0.632 (0.486, 0.823)**	0.002	**0.528 (0.406, 0.688)**	0.000	**0.529 (0.383, 0.73)**	0.001
*p* for trend		0.001		<0.001		0.002	
VB12	25.09–2952.00						
Q1	25.09–265.68	1		1		1	
Q2	266.40–352.00	0.726 (0.511, 1.032)	0.086	0.714 (0.502, 1.017)	0.077	0.75 (0.472, 1.189)	0.239
Q3	352.80–467.10	**0.654 (0.49, 0.872)**	0.008	**0.656 (0.487, 0.885)**	0.012	0.738 (0.513, 1.062)	0.121
Q4	467.90–2952.00	**0.445 (0.324, 0.611)**	0.000	**0.456 (0.324, 0.64)**	0.000	**0.54 (0.36, 0.809)**	0.009
*p* for trend		<0.001		<0.001		0.005	
VB9	5.40–144.30						
Q1	5.40–20.60	1		1		1	
Q2	20.80–29.00	**0.683 (0.522, 0.894)**	0.010	**0.665 (0.503, 0.879)**	0.010	**0.668 (0.485, 0.92)**	0.025
Q3	29.20–40.50	**0.538 (0.395, 0.733)**	0.001	**0.535 (0.381, 0.753)**	0.002	**0.559 (0.357, 0.878)**	0.022
Q4	40.80–144.30	**0.52 (0.377, 0.716)**	0.000	**0.492 (0.359, 0.676)**	0.000	**0.537 (0.364, 0.793)**	0.006
*p* for trend		0.002		0.004		0.023	

All estimates accounted for complex survey designs. Model 1 contains only independent variables; model 2 was additionally adjusted for gender, age, ethnicity, FIPR and education level; and model 3 was further adjusted for the disease history (hypertension, high cholesterol and diabetes). Those results in bold had statistical significance. Q1, Q2, Q3, and Q4 indicated the 1st, 2nd, 3rd, and 4th quartiles across the lowest to the highest concentrations of circulating vitamins.

### Dose-response relationships between serum vitamin levels and non-alcoholic fatty liver disease

**FIGURE 3:**
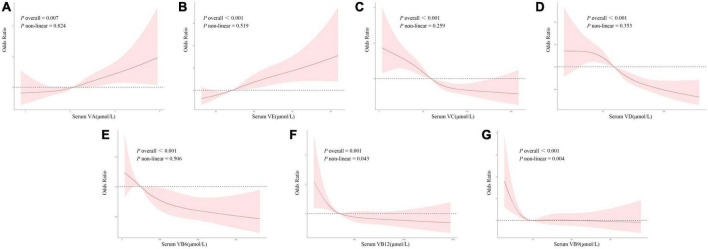
**(A–G)** Dose–response relationships between serum vitamin levels and the risk of NAFLD with restricted cubic spline model. The model was adjusted for gender, age, ethnicity, FIPR, education level, and disease history (hypertension, high cholesterol, and diabetes).

Dose–response curves for the relationships between all studied vitamins and the risk of NAFLD were presented in Figure 3, which showed similar directions to those curves in the single-vitamin logistic regression model.

Figure 3A showed the dose–response relationship between serum VA level and NAFLD. There was a positive linear correlation between NAFLD and VA (*p* overall = 0.007, *p* non-linear = 0.824). Lower serum VA level was uncorrelated with NAFLD risk. However, the risk of NAFLD increased linearly as serum VA levels elevated when the latter exceeded a certain threshold.

Figure 3B showed the dose–response relationship between serum VE level and NAFLD. There was a positive linear correlation between NAFLD and VE (*p* overall < 0.001, *p* non-linear = 0.519). Lower serum VE level could be protective against NAFLD (OR < 1), but its protective effect weakened with elevated levels. The risk of NAFLD increased as serum VE levels elevated when the latter exceeded a certain threshold (OR > 1).

Figure 3C showed the dose–response relationship between serum VC level and NAFLD. There was a negative linear correlation between NAFLD and VC (*p* overall < 0.001, *p* non-linear = 0.259). A lower serum VC level could be a risk factor for NAFLD (OR > 1), but its risk effect weakened with an elevated level. The risk of NAFLD decreased as serum VC levels elevated when the latter exceeded a certain threshold (OR < 1). However, serum VC was uncorrelated with NAFLD risk when it was above a limited level.

Figure 3D showed the dose–response relationship between serum VD level and NAFLD. There was a negative linear correlation between NAFLD and VD (*p* overall < 0.001, *p* non-linear = 0.353). Lower serum VD level could be a risk factor for NAFLD (OR > 1), but its risk effect weakened with elevated levels. The risk of NAFLD decreased consistently as serum VD levels elevated when the latter exceeded a certain threshold (OR < 1).

Figure 3E showed the dose–response relationship between serum VB6 level and NAFLD. There was a negative linear correlation between NAFLD and VB6 (*p* overall < 0.001, *p* non-linear = 0.506). Its dose–response trend was similar to that of VD.

Figure 3F showed the dose–response relationship between serum VB12 level and NAFLD. There was a non-linear correlation between NAFLD and VB12 (*p* overall = 0.001, *p* non-linear = 0.043). Lower serum VB12 level could be a risk factor for NAFLD (OR > 1), but its risk effect weakened with elevated levels. However, serum VB12 was uncorrelated with NAFLD risk when it was above a limited level.

Figure 3G showed the dose–response relationship between serum VB9 level and NAFLD. There was a non-linear correlation between NAFLD and VB9 (*p* overall <0.001, *p* non-linear = 0.004). Its dose–response trend was similar to that of VB12.

### Serum multivitamin levels and the risk of non-alcoholic fatty liver disease

**FIGURE 4:**
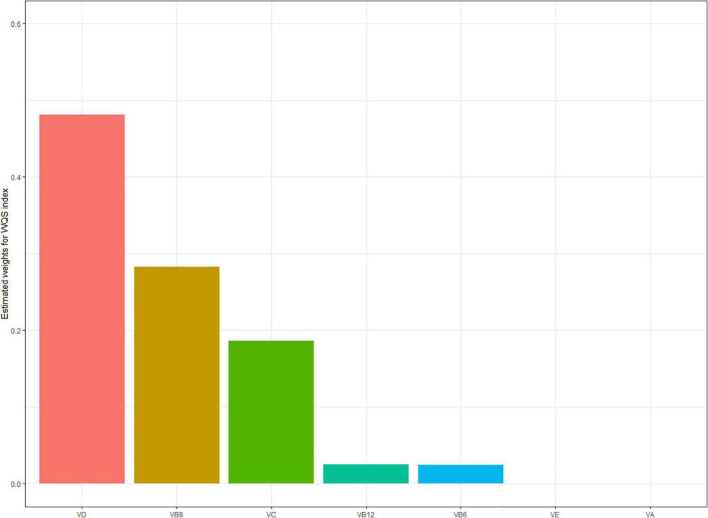
Estimated weights assigned to each vitamin level with the WQS regression model in a negative direction. Model adjusted for gender, age, ethnicity, FIPR, education level, and disease history (hypertension, high cholesterol, and diabetes).

Table 4 showed the results from rough and fully adjusted models with WQS regression. We found the indices in WQS were significantly associated with NAFLD. In the fully adjusted models, each increment of the index was associated with 41.5% lower odds of NAFLD (OR = 0.585, 95% CI: 0482–0.712). We also observed the highest weights in WQS model: 0.48 for VD, 0.28 for VB9, and 0.19 for VC in negative correlation. VE and VA were observed in positive correlation (Figure 4).

**TABLE 4:** Overall effects of the multivitamins estimates and 95% confidence interval.

Model	OR	*P*
Model 1	0.580 (0.508, 0.662)	<0.001
Model 2	0.527 (0.447, 0.622)	<0.001
Model 3	0.585 (0.482, 0.712)	<0.001

All estimates accounted for complex survey designs. Model 1 included only independent variables; model 2 was additionally adjusted for gender, age, ethnicity, FIPR, and education level; and model 3 was further adjusted for the disease history (hypertension, high cholesterol, and diabetes).

## Discussion

To date, most population-based research studies focused on the relationship between single vitamin and the risk of NAFLD (9, 18, 19). A few research studies explored their dose–response relationship. There was no reported evidence for the correlations between multivitamins and NAFLD risk. People normally encounter mixed exposures rather than a single exposure due to multivitamins contained in daily food. Unlike previous studies, in this cross-sectional study of a nationally representative sample, we explored for the first time the associations between serum multivitamin levels and the risk of NAFLD as well as their dose–response relationships through comprehensive analyses with multiple novel statistical approaches. We found that serum levels of VC, VD, VB6, and VB9 were negatively correlated with the risk of NAFLD, while serum levels of VA and VE were positively correlated with the risk of NAFLD. We also observed linear correlations of those five vitamins with the risk of NAFLD from the dose–response curves. WQS regression suggested significant associations between serum multivitamin levels and reduced risk of NAFLD, with major contributors of VD, VB9, and VC. Our findings suggested that serum multivitamin levels were significantly associated with the risk of NAFLD.

### Vitamin A and the risk of non-alcoholic fatty liver disease

As a lipid-soluble vitamin and an antioxidant, VA (retinol) existed abundantly in animals, especially in fish. Interestingly enough, VA intake might not guarantee any antioxidative and/or protective effects (20). Instead, long-term intake of VA would elevate the levels of alkaline phosphatase, triacylglycerol, and cholesterol (21), which were somehow associated with the risk of NAFLD. Based on the dose–response relationship curves, we suggested that there was a linear positive correlation between serum VA level and the risk of NAFLD. VA played an important role in both metabolic regulation and hepatic stellate cell activation, so it might be a critical factor during the exploration of NAFLD pathogenesis (22). Previous studies proposed some evidence for the association between VA and NAFLD. Similar to our findings, Bahcecioglu et al. suggested that patients with non-alcoholic steatohepatitis (NASH) or simple hepatic steatosis have elevated serum VA levels compared with healthy individuals (23). According to a prospective longitudinal study including 2,658 participants (24), higher serum VA level was associated with the progression of NAFLD, which was possibly attributed to increased triglycerides, insulin resistance, serum retinol-binding protein 4, and BMI. However, some studies proposed different conclusions. Botella-Carretero et al. suggested that serum VA level was negatively correlated with BMI for morbidly obese people as well as the level of serum transaminase for NAFLD patients (25). A study suggested that there was a significant association between low retinol levels and insulin resistance (26). Those discrepancies might be due to the differences in overall serum VA levels in the included population. According to the dose–response curves, a lower serum VA level was protective against NAFLD, while higher serum VA level became a risk factor for NAFLD. Therefore, we suggested that maintaining a proper serum VA level might be good for health.

### Vitamin C and the risk of non-alcoholic fatty liver disease

We found that serum VC level was negatively correlated with the risk of NAFLD, as well as the most important contributor to the prevalence of NAFLD in U.S. adults. Vitamin C, also known as ascorbic acid, was a water-soluble vitamin and existed in a variety of vegetables and fruits. Wei et al. (27) found that the risk of NAFLD was decreased by 0.71 times in the highest quartile of dietary vitamin C intake compared with the lowest quartile, which was similar to our findings. More and more epidemiologic studies proposed that serum VC levels had a significant negative correlation with hepatic steatosis and hepatic fibrosis (28, 29). According to a prospective double-blinded randomized controlled trial, oral VC supplements could significantly improve liver function and glucose metabolism as well as guarantee intestinal microbial diversity and adiponectin concentration for patients with NAFLD (30). A suitable intake of VC could alleviate hepatic fibrosis for patients with NASH (31). And as an antioxidant, VC could scavenge free radicals, enhance the activity of manganese superoxide dismutase (SOD) and glutathione peroxidase (GPx), improve the secretion and expression of adiponectin, and lower the levels of low-density lipoprotein cholesterol (LDL-C) and triglycerides (TG) (32–35), which provided possible explanations for that VC could reduce the risk of NAFLD.

### Vitamin D and the risk of non-alcoholic fatty liver disease

Vitamin D, a secosteroid, had an influence not only on calcium homeostasis but also on cell differentiation and proliferation, immune modulation, and inflammatory response. We found that there was a negative linear correlation between serum VD level and the risk of NAFLD. According to a cross-sectional study including 16,190 participants (36), serum VD level had a significant negative correlation with liver enzyme, insulin resistance, and the components of metabolic syndrome, which was similar to our findings. The elevated serum VD level might alleviate inflammation/steatosis of the liver and improve insulin sensitivity by activating liver macrophage vitamin D receptors (VDR) (37). However, some pieces of evidence for the association between VD deficiency (VDD) and the risk of NAFLD were still controversial. Animal studies suggested that VDD would exacerbate NAFLD by activating Toll-like receptors (TLRs). Moreover, VDD would result in insulin resistance, up-regulation of hepatic inflammatory and oxidative stress genes, and higher hepatic resistin gene expression (38). On the contrary, a 16-week RCT study (39) reported that VD supplements in non-Western VDD immigrants with prediabetes failed to improve insulin sensitivity or β-cell function or change the incidence of metabolic syndrome. One possible explanation would be that the change in insulin sensitivity after the VD supplement was influenced by the single nucleotide polymorphisms (SNPs) of the VDR gene (40). Therefore, SNPs of the VDR gene should be considered when concluding that the VD supplement was beneficial to NAFLD.

### Vitamin B6 and the risk of non-alcoholic fatty liver disease

VB6 was a key cofactor in the metabolism of amino acids, glucose, and fat. Similar to VC and VD, VB6 was also negatively correlated with the risk of NAFLD. Federico et al. found that patients with NASH had a less daily intake of VB6 than those in the control group (41). A prospective clinical study suggested that an oral VB6 supplement could significantly ameliorate liver fat accumulation in patients with NAFLD (42). Moreover, Lin et al. proposed that a borderline VB6 deficit was associated with the increased risk of dyslipidemia and coronary artery disease (43). The deficiency of VB6 resulted in homocysteine (Hcy) accumulation. Hcy would induce protein misfolding in the endoplasmic reticulum (ER), triggering a stress response in the ER. Then ER stress drives *de novo* lipogenesis by activating the transcription factor sterol response element-binding protein 1c (SREBP-1c), resulting in NAFLD development (44). VB6 could prevent insulin resistance, endothelial dysfunction, and liver fat accumulation (45), which were possible underlying mechanisms of VB6 to reduce the risk of NAFLD.

### Vitamin B9 and the risk of non-alcoholic fatty liver disease

VB9, also known as folate, is a water-soluble vitamin widely distributed in green leafy vegetables such as spinach, beet, and kale. The dose–response curve showed that serum VB9 was non-linearly correlated with NAFLD risk and would increase the incidence of NAFLD when staying at a lower level. Similarly, Xia et al. (8) suggested that serum folate level was negatively correlated with the grade of hepatic steatosis and liver fat content, and low serum folate level was an independent risk factor for NAFLD. The liver was an essential organ for storage and metabolism of folate. More and more research studies confirmed that folate was closely associated with lipid metabolism. Folate deficiency might depress phospholipid *N*-methylation in the liver and reduce *de novo* phosphatidylcholine synthesis, resulting in TG accumulation in the liver (46), which was further verified in animal experiments by Pogribny et al. (47). Moreover, folate deficiency would accelerate the synthesis of hepatic lipid by inducing related genes, resulting in hepatic steatosis (48). It was noteworthy that excessive high serum folate levels might increase the risk of cognitive disorder and insulin resistance (49). Maintaining a proper serum folate level might be good for health.

### Vitamin E and the risk of non-alcoholic fatty liver disease

In this study, serum VE level had a significant positive correlation with the risk of NAFLD. With each increment of the VE level, the risk of NAFLD elevated by approximately 1.9% (95% CI: 1.1–1.2%). Jeon et al. suggested that serum VE level was positively associated with the prevalence of NAFLD, which was similar to our findings (50). Alpha-tocopherol (VE) was identified as one of the predictors of MRI-determined liver fat (51). A cross-sectional study on Swedish adults proposed that serum VE level was positively associated with serum cholesterol and obesity (52). Waniek et al. (53) suggested that alpha-tocopherol level was positively associated with high triglycerides and low high-density lipoprotein cholesterol (HDL-C) levels. Moreover, serum VE levels played important roles in visceral adipose tissue and metabolic syndrome. Animal experiments indicated that mice with NAFLD had impaired liver metabolism and gene response of alpha-tocopherol (54). All these studies provided evidence that serum VE level was positively associated with the risk of NAFLD.

### Multi-vitamins and the risk of non-alcoholic fatty liver disease

People were exposed to a variety of vitamins in daily life due to different dietary structures or nutritional components in food. It was necessary to explore the effect of multi-vitamins on health. In this study, we employed the WQS regression model to explore the association between serum levels of multivitamins and the risk of NAFLD. Results indicated that higher serum levels of multivitamins are associated with reduced NAFLD risk, despite different influences and weights of a single vitamin. There was no study exploring the relationship between multivitamins and NAFLD. It was incorrect to include all studied vitamins in a single generalized linear regression model, which might distort the results due to the correlation that exists among vitamins (55). The WQS was a novel model to explore the influences of multivitamins on health, by considering highly correlated vitamins. Based on the weights empirically determined by bootstrap sampling, we used WQS to examine the whole-body burden with serum levels of multivitamins. The WQS enabled us to encompass the complex serum levels of multivitamins in the real world. In this study, VD, VB9, and VC were weighted highly. More prospective cohort and large-population studies are necessary to determine the contributions and mechanism of serum multivitamin levels to NAFLD.

### Strengths and limitations

Strengths: We employed, in this study, for the first time both logistic regression and WQS regression models that enabled us to systematically assess the association between serum multivitamin levels and the risk of NAFLD, as well as to explore the dose–response relationship between single vitamin and the risk of NAFLD. Moreover, this study covered a wide range of participants with good sample representativeness. We also adjusted important covariates in our models, including demographic characteristics and disease history.

Limitations: First, serum levels of vitamins were tested only once at baseline, which might not represent a long-term status. Second, the diagnosis of NAFLD was based on the FLI model, instead of a gold standard in histology. Third, although we adjusted multiple covariates in our study, there might be other potential confounders such as dietary intakes, physical activities, and metabolic syndrome. And last, our work was based on a cross-sectional study and could not confirm the cause-and-effect relationships between the serum vitamin level and the risk of NAFLD. More large-scale and prospective cohort studies should be encouraged in the future.

## Conclusion

This cross-sectional study explored the association between serum levels of 7 common vitamins and the risk of NAFLD. Among U.S. adults, high serum levels of VC, VD, VB6, and VB9 and low serum levels of VA/VE were associated with reduced risk of NAFLD. Among all the 7 kinds of vitamins, VD was weighted highest and the most important. Proper serum levels of multivitamins contributed to a lower risk of NAFLD. Our findings suggested a novel perspective to explore the association between multivitamins and NAFLD risk. Further studies are still required to reveal the underlying mechanisms of multivitamins to NAFLD.

## Data availability statement

The original contributions presented in this study are included in the article/Supplementary material, further inquiries can be directed to the corresponding author/s.

## Ethics statement

The protocol of NHANES was reviewed and approved by the Research Ethics Review Board of the National Center for Health Statistics. The patients/participants provided their written informed consent to participate in this study.

## Author contributions

HP and WL conceived of the study. HP, MW, and LP conducted the data analysis and drafted the manuscript. ZC, ZY, and QC drafted the manuscript. YL and YW visualized the result. All authors edited the manuscript, read and approved the final manuscript.
